# SAMD13 serves as a useful prognostic biomarker for hepatocellular carcinoma

**DOI:** 10.1186/s40001-023-01347-5

**Published:** 2023-11-15

**Authors:** Wonbeak Yoo, Seokho Kim, KyungHee Noh

**Affiliations:** 1 Personalized Genomic Medicine Research Center, Korea Research Institute of Bioscience and Biotechnology (KRIBB), Daejeon, 34141 Republic of Korea; 2 Department of Health Sciences, The Graduate School of Dong-A University, Busan, 49315 Republic of Korea; 3 Department of Medicinal Biotechnology, College of Health Sciences, Dong-A University, 37, Nakdong-daero 550 beon-gil, Saha-gu, Busan, 49315 Republic of Korea; 4 Bionanotechnology Research Center, Korea Research Institute of Bioscience and Biotechnology, Daejeon, 34141 Republic of Korea; 5 Department of Nanobiotechnology, University of Science and Technology (UST), Daejeon, 34141 Republic of Korea

**Keywords:** SAMD13, Hepatocellular carcinoma, Prognosis, Epigenetic regulation, Drug-resistance

## Abstract

Hepatocellular carcinoma (HCC) is the most common form of liver cancer and the 5-year relative overall survival (OS) rate is less than 20%. Since there are no specific symptoms, most patients with HCC are diagnosed in an advanced stage with poor prognosis. Therefore, identifying novel prognostic biomarkers to improve the survival of patients with HCC is urgently needed. In the present study, we attempted to identify SAMD13 (Sterile Alpha Motif Domain-Containing Protein 13) as a novel biomarker associated with the prognosis of HCC using various bioinformatics tools. SAMD13 was found to be highly expressed pan-cancer; however, the SAMD13 expression was significantly correlated with the worst prognosis in HCC. Clinicopathological analysis revealed that SAMD13 upregulation was significantly associated with advanced HCC stage and high-grade tumor type. Simultaneously, high SAMD13 expression resulted in association with various immune markers in the immune cell subsets by TIMER databases and efficacy of immunotherapy. Methylation analysis showed SAMD13 was remarkably associated with prognosis. Furthermore, a six-hub gene signature associated with poor prognosis was correlated with the cell cycle, transcription, and epigenetic regulation and this analysis may support the connection between SAMD13 expression and drug-resistance. Our study illustrated the characteristics of SAMD13 role in patients with HCC using various bioinformatics tools and highlights its potential role as a therapeutic target and promising biomarker for prognosis in HCC.

**Supplementary Information:**

The online version contains supplementary material available at 10.1186/s40001-023-01347-5.

## Introduction

Hepatocellular carcinoma (HCC) has one of the highest morbidity and mortality globally and the incidence of HCC is significantly increased annually. HCC ranked second and third in major causes of cancer-related deaths due to the complexity, heterogeneity, and high recurrence following surgical resection [1, 2]. In patients with early stage HCC, surgical or percutaneous resection is recommended as a first-line treatment option; however, the recurrence rate is around 50% 5-year post-surgery [3, 4]. The prognostic markers play an important role in monitoring tumor recurrence or metastasis and judging prognosis [5]. Therefore, identification of specific prognostic biomarker of HCC helps to provide a great opportunity to improve the prognosis in patients.

SAMD13 gene is cytogenetically located on the short (p) arm of chromosome 1, at position 31.1 (1p31.1) and one of the sterile alpha motif domain-containing (SAMD)-proteins. The SAMD is the most common protein interaction module which has been implicated in diverse biological roles for cellular processes, such as binding to self- or other SAMD, non-SAMD, RNA, DNA, and even lipids [6–12]. A previous study revealed that SAMD13 was downregulated on the micropapillary area in invasive micropapillary carcinoma (IMPC) [13]. This study suggested that SAMD13 expression is relevant to the pathological features and immune microenvironment in IMPC; however, the expression profile and biological role of SAMD13 in hepatocellular carcinoma remain totally unknown.

This study aimed to explore the expression profile and prognostic effects of SAMD13 using pan-cancer database and various bioinformatics in patients with HCC. Furthermore, we examined the relationship between SAMD13 expression and clinicopathological criteria of The Cancer Genome Atlas Liver Hepatocellular Carcinoma (TCGA–LIHC) using UALCAN. Subsequently, we performed methylation profiling analyses to predict the overall survival rate of patients with HCC. The correlation between SAMD13 and tumor-infiltrating immune cells in the tumor microenvironment of HCC was also determined. Networks and functional enrichment analyses were further performed to evaluate whether SAMD13 expression is associated with transcriptional and epigenetic regulation and its related genes in clinical features. Finally, based on the functional enrichment analyses, the importance of SAMD13 gene for drug resistance and its value as targets for novel drug development using GEO data set was validated. Taken together, our results provided a better understanding of clinical significance of SAMD13 in HCC, and prognostic assessment implication in patients with HCC.

## Materials and methods

### Data acquisition and processing

UALCAN database is an open-access web for analyzing cancer OMICS data based on tumors and normal samples from TCGA, MET500, CPTAC, and CBTTC [14]. Here, the UALCAN was used to compare the expression levels of SAMD13 in pan-cancer, including HCC and clinical data, as well as the associations between them. GEPIA and GEPIA2 is a web tool for gene expression profiling and interactive analyses based on TCGA and The Genotype-Tissue Expression (GTEx) data [15]. This database was used to access overall survival (OS) and disease-free survival (DFS) based on the expression of SAMD13, and the further association between this gene and the expression of immune-related marker genes was verified.

### Microarray data

The TCGA–LIHC (HCC) data set was used as the training set, while three other independent data sets (GSE22058, GSE25097, and GSE45436) and Gene Expression database of Normal and Tumor tissues 2 (GENT2) (which is web-based tool from public gene expression data sets) were used as validation sets. Gene expression profiling data sets, including GSE22058 (97 non-tumor and 100 tumor), GSE25097 (6 normal, 40 cirrhosis, and 268 tumor), and GSE45436 (39 non-tumor and 95 tumor), and chemo-resistant gene profiling data sets, including GSE54175 (cisplatin- or doxorubicin-resistant subline), GSE93595 (anti-angiogenic therapy (JNJ-28841072)-resistant subline), GSE121153 (sorafenib-resistant subline), GSE125180 (doxorubicin-resistant subline), GSE109211 (clinical parameters from responders and non-responders to sorafenib in patients with HCC) were downloaded from the gene expression omnibus (GEO, https://www.ncbi.nlm.nih.gov/geo/). cBioportal for Cancer Genomics database which analyzes multi-omics data from The Cancer Genome Atlas was used for analyzing genetic alteration for SAMD13 gene in a liver study (TCGA, PanCancer Atlas) [16]. The KM–plotter database was used for survival curves in various cancers [17]. Hazard ratios (HR) and *p* values (from the log-rank test) were calculated online.

### Characterization of tumor microenvironment in HCC

Human Liver Browser and Single-cell Atlas in Liver Cancer (scAtlasLC) were used to characterize SAMD13 expression of single-cell transcriptomic profiles in HCC [18, 19]. The Tumor Immune Estimation Resource database (TIMER 2.0) used analysis and visualization of immune infiltrates from TCGA in TIMER Database [20]. In addition to exploring the associations of HCC-infiltrating immune cells with SAMD13 gene expression, the degree of immune infiltration of B cell, CD8 + T cell, CD4 + T cell, macrophages, neutrophil, and dendritic cell (DC) was analyzed in TIMER database. The strength of correlations evaluated by the purity-adjusted partial Spearman’s rho value and estimated statistical significance in TIMER 2.0.

### Comprehensive analysis of SAMD13 methylation

MethSurv database could provide the initial assessment of DNA methylation biomarkers using TCGA [21]. In this study, single CpG methylation of SAMD13 and Heatmap analysis were performed in patients with HCC. Next, Shiny Methylation Analysis Resource Tool (SMART) [22] was used to analyze differential methylation by each SAMD13 probe and Spearman’s correlation between methylation level and mRNA level. The CpG-aggregated methylation values were measured by mean (β values). To characterize the methylation patterns, the significant CpGs were classified according to their functional roles in genomic locations, such as promoters within 1,500 bps of a transcription start site (TSS) (TSS1500); within 200 bps of a TSS (TSS200); 5’ untranslated regions (5'UTR); first exon (1stExon); body (non-promoter); 3'UTR (non-promoter).

### Functional and pathway enrichment analysis

The 22 genes with the strongest correlation with SAMD13 were selected using Pathway Commons [23] and this allowed the creation of a protein–protein interaction (PPI) network for SAMD13 genes as well as binding and targeted genes. To determine the functional and pathway meaning, cellular component (CC), molecular function (MF), biological process (BP), and reactome pathway (RP) for 22 selected genes forming a cluster with SAMD13 was performed using geneontology.org [24]. Functional enrichment analyses of Gene Ontology (GO) terms were performed using Fisher's Exact test and adjusted by a False Discovery Rate (FDR) correction for multiple testing in pantherdb.org (http://pantherdb.org/).

### SAMD13 Immunohistochemistry staining

SAMD13 protein expression levels in normal and HCC tissues were reviewed in the Human Tissue Atlas which is an online tool for genome-wide analysis (http://www.proteinatlas.org/) [25].

### Cell lines and cell culture

Huh7, HepG2, SK-Hep1 cells were purchased from the American Type Culture Collection. SNU475 and SNU449 cells were Korean Cell Line Bank (Seoul, Republic of Korea). Huh7, HepG2, SK-Hep1 Cells were maintained in Dulbecco’s modified Eagle’s medium containing 10% FBS (GIBCO, Grand Island, NY, USA).SNU475 and SNU449 cells were maintained in RPMI1640 medium containing 10% FBS in a humidified incubator with 5% CO_2_ at 37 °C. HepaRG cells were cultured in William’s E medium supplemented with 10% of FBS, 5 µg/mL insulin, 2‐mM Glutamax, 1% penicillin–streptomycin, and 50 µM hydrocortisone hemisuccinate (Sigma-Aldrich).

### Real-time quantitative reverse transcription-polymerase chain reaction (qRT-PCR) analysis

Total RNA was isolated using a TRIzol reagent-based kit (Intron Biotech, Seongnam-Si, Republic of Korea). Reverse transcription was performed using the SuperScript IV First-Strand Synthesis System for RT-PCR (Thermo Fisher Scientific, Waltham, MA, USA) according to the manufacturer’s protocol. cDNA was amplified with specific primers and SYBR Premix Ex Taq (Takara Bio, Otsu, Shiga, Japan and Agilent Technologies, Santa Clara, CA, USA). The SAMD13 mRNA levels were normalized to the amount of GAPDH mRNA. qPCR was performed according to the manufacturer’s instructions (Applied Biosystems, Foster City, CA, USA, and Agilent Technologies). Relative quantification of gene expression was performed using the 2-ΔΔCT method. The primer pair used for SAMD13 (forward: 5’-CAAGGAAAATGGCTCTGTCGGTG-3’; reverse: 5’-AGCTTGCTCCTCAAATCCCACG-3’) and GAPDH (forward: 5’-AACGGGAAGCTTGTCATCAATGGAAA-3’; reverse: 5’- GCATCAGCAGAGGGGGCAGAG-3’).

### Statistical analysis

The statistical analysis was calculated automatically based on the online database above. Student's *t* test implemented by GraphPad Prism (Ver7).Correlations were analyzed by Spearman and Pearson’s correlation. *p* < 0.05 was considered statistically significant.

## Results

### Impact of expression and prognosis of SAMD13 in various human cancers

**Fig. 1:**
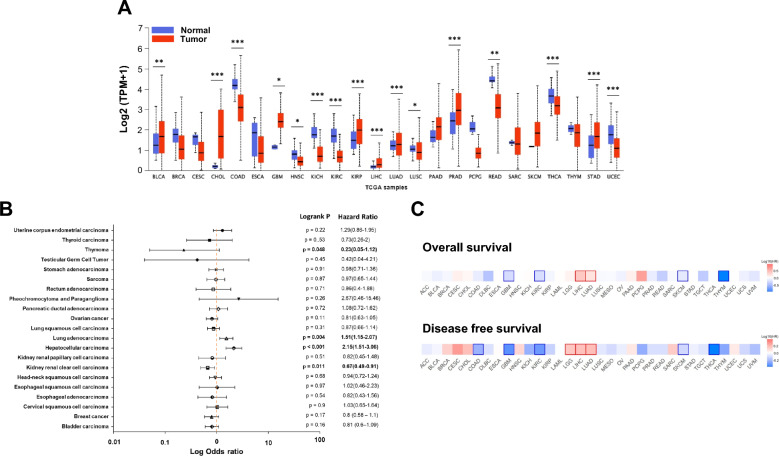
Impact of SAMD13 expression on prognosis in various human cancers. **A** Expression levels of SAMD13 in UALCAN database. **B**, **C** Pan-cancer analysis of the prognostic values of SAMD13. (*p < 0.05, **p < 0.01, ***p < 0.001). *ACC* Adrenocortical carcinoma, *BLCA* Bladder Urothelial Carcinoma, *BRCA* Breast invasive carcinoma, *CESC* Cervical squamous cell carcinoma and endocervical adenocarcinoma, *CHOL* Cholangiocarcinoma, *COAD* Colon adenocarcinoma, *DLBC* Lymphoid Neoplasm Diffuse Large B-cell Lymphoma, *ESCA* Esophageal carcinoma, *GBM* Glioblastoma multiforme, *HNSC* Head and Neck squamous cell carcinoma, *KICH* Kidney Chromophobe, *KIRC* Kidney renal clear cell carcinoma, *KIRP* Kidney renal papillary cell carcinoma, *LAML* Acute Myeloid Leukemia, *LGG* Brain Lower Grade Glioma, *LIHC* Liver hepatocellular carcinoma, *LUAD* Lung adenocarcinoma, *LUSC* Lung squamous cell carcinoma, *MESO* Mesothelioma, *OV* Ovarian serous cystadenocarcinoma, *PAAD* Pancreatic adenocarcinoma, *PCPG* Pheochromocytoma and Paraganglioma, *PRAD* Prostate adenocarcinoma, *READ* Rectum adenocarcinoma, *SARC* Sarcoma, *SKCM* Skin Cutaneous Melanoma, *STAD* Stomach adenocarcinoma, *TGCT* Testicular Germ Cell Tumors, *THCA* Thyroid carcinoma, *THYM* Thymoma, *UCEC* Uterine Corpus Endometrial Carcinoma, *UCS* Uterine Carcinosarcoma, *UVM* Uveal Melanoma

To explore the expression pattern of SAMD13between tumor and normal tissues in various types of cancer, we examined the SAMD13 differences using the UALCAN database. The comparison of expression level between each type of normal and cancer cells revealed that SAMD13 increased in breast invasive carcinoma, cholangiocarcinoma, glioblastoma multiforme, kidney renal papillary cell carcinoma, liver hepatocellular carcinoma, lung adenocarcinoma, prostate adenocarcinoma, and stomach adenocarcinoma, while decreased in colon adenocarcinoma, head and neck squamous cell carcinoma, kidney chromophobe, rectum adenocarcinoma, thyroid carcinoma, and uterine corpus endometrial carcinoma (Fig. 1A). To validate these findings, we further examined the expression levels of SAMD13 using the GENT2 portal. As shown in Additional file 1: Figure S1, SAMD13 was upregulated in blood, brain, liver, and bone cancers, while downregulated in colon, breast, kidney, head and neck, tongue, thyroid, adrenal gland, pharynx, testis, and endometrial cancers. To assess the prognostic significance of SAMD13 expression in various types of cancers, log-rank test was performed using pan-cancer RNA-seq in KM-plotter. Among these 21 types of cancer, four were significantly associated with the prognosis of all cancers (Fig. 1B). High mRNA expression level of SAMD13 were observed to be significantly associated with worse overall survival in lung adenocarcinoma (HR, 1.51; 95% CI 1.15–2.07; *p* = 0.004) and hepatocellular carcinoma (HR, 2.15; 95% CI 1.15–3.06; *p* < 0.001), whereas thymoma (HR, 0.23; 95% CI 0.05–1.12; *p* = 0.048) and kidney renal clear cell carcinoma (HR, 0.011; 95% CI 0.49–0.91; *p* = 0.011) were significantly associated better prognosis. In addition, these observations were validated by GEPIA2 in pan-cancer database. As shown in Fig. 1C, high SAMD13 expression is associated with a poorer overall survival LIHC and LUAD, while low expression of SAMD13 indicated better prognosis in THYM. When analyzing disease-free survival, the high expression of SAMD13 is associated with a worse prognosis in LGG, LICH, and LUAD, while the low expression of SAMD13 indicated better prognosis in COAD, GBM, KIRC, and THCA.

### Validation and survival analysis of SAMD13 in HCC

**Fig. 2:**
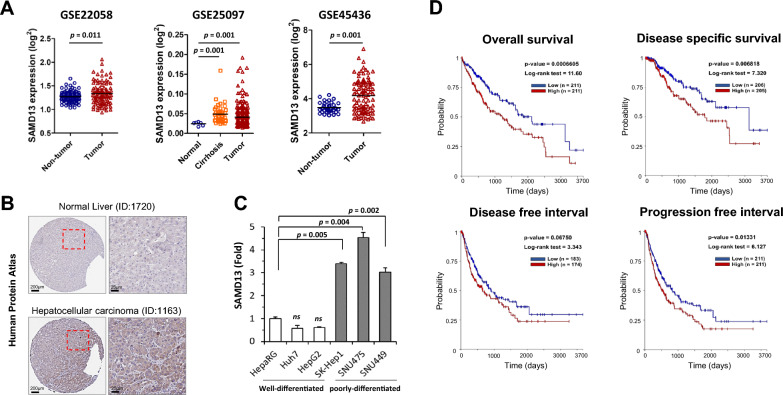
Clinical signification of SAMD13 expression for predicting the prognosis of HCC. **A** Validation of SAMD13 expression in cohorts from the GEO data set. **B** Representative immunohistochemistry images of SAMD13 in HCC tissues and normal tissues (Human Protein Atlas). **C **Up-regulation of SAMD13 in poorly differentiated HCC cell lines. qRT-PCR were performed in six different HCC cell lines. **D** Survival outcomes based on Kaplan–Meier survival analysis of patients from The Cancer Genome Atlas (TCGA) according to SAMD13 expression levels; *p* values derived from the log-rank test are indicated in each comparison

**Fig. 3:**
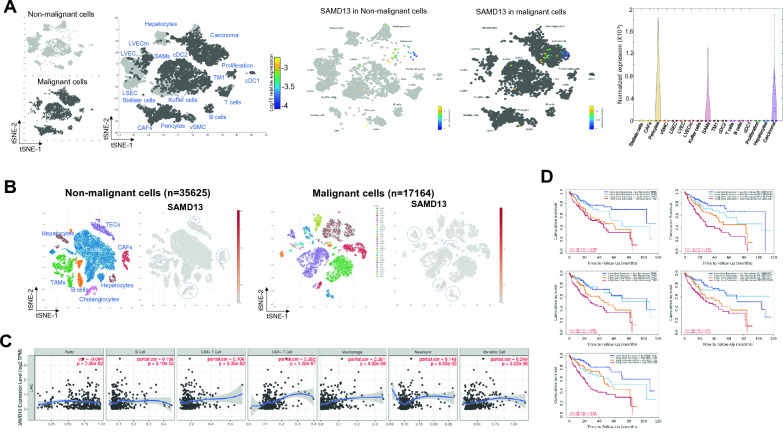
Characterization of SAMD13expression on TME in HCC. The expression of the SAMD13 in non-malignant and malignant cells in HCC was analyzed by Human Liver Browser (**A**) and scAtlasLC (**B**). **C** Correlation analysis of SAMD13 expression with immune infiltration level in HCC. **D** Comprehensive prognostic value of SAMD13 expression and neutrophil, macrophage, macrophage M0, macrophage M2, and MDSC infiltration levels based on the TIMER algorithm. *CAFs* cancer-associated fibroblasts, *cDC* conventional dendritic cell, *LSEC* liver sinusoidal endothelial cell, *LVEC* lymphatic vascular endothelial cell, *LVECm* malignant lymphatic vascular endothelial cell, *SAMs* scar-associated macrophages, *TM* tissue monocytes, *vSMC* vascular smooth muscle cell, *TAMs* tumor associated macrophages, *TECs* tumor-associated endothelial cells

Since SAMD13 gene expression was not only upregulated in various carcinomas but also linked to worst prognosis in HCC, we further performed validation on additional three independent GEO data sets. As expected, these GEO data sets also revealed the significant augmentation of SAMD13 mRNA expression in the tumor group compared to their normal/non-tumor group. Interestingly, increased mRNA expression of SAMD13 was also revealed in the cirrhosis group at GSE25097 (Fig. 2A). Next, this trend was evaluated at the protein level between normal liver and HCC tissues, and as a result, the immunohistochemical data show that SAMD13 expression was higher than that compared to normal tissue (Fig. 2B).To further validate the differential expression of SAMD13, mRNA expression of SAMD13 was conducted on 1 immortalized hepatic cell line (HepaRG), 2 well-differentiated HCC cell lines (Huh7 andHepG2), and 3 poorly differentiated HCC cell lines (SK-Hep-1, SNU475, and SNU449). As shown Fig. 3C, the SAMD13 expression were increased in poorly differentiated HCC cells relative to well-differentiated HCC cells suggesting that SAMD13 may be acted HCC tumors to more aggressive tumor phenotypes. To better understand the relationship between SAMD13 expression and prognosis in HCC, we investigated the correlation between SAMD13 expression and overall survival (OS), disease specific survival (DSS), disease free interval (DFI), and progression free interval (PFI). As shown in Fig. 2D, high expression of SAMD13 was significantly associated with shorter OS (log-rank test = 11.6; *p* = 0.0006605), DSS (log-rank test = 7.32; *p* = 0.006818), and PFI (log-rank test = 6.127; *p* = 0.01331); meanwhile, DFI was correlated with shorter tendency (log-rank test = 3.343; *p* = 0.0675).

### Clinicopathological features and SAMD13 expression

We then determined a relationship between clinicopathological features and SAMD13 expression using TCGA data through UALCAN in HCC, and summarized in Table1. According to tumor stage, SAMD13 expression were significantly upregulated in HCC group classified as stage I to stage III compared to the corresponding normal group. Based on tumor grade, a significant increase in SAMD13 levels was observed in the HCC group in all tumor grades. In addition, upregulation of SAMD13 expression was observed in patients with HCC and fibrolamellarcarcinoma types. In addition, SAMD13 expression was statistically significant in all other categories of patients with HCC, including gender, age, and race. These results suggest that SAMD13 was significantly correlated with clinical parameters in patients with HCC.

**Table 1:** Association between clinical characteristic parameters and the expression SAMD13 of HCC patients in TCGA

	Expression of SAMD13 (Transcript per million) median value	*p* value
Sex (*n* = 412)		
Normal (*n* = 50)	0–0.363 (0.142)	
Male (*n* = 245)	0–1.311 (0.229)	< 0.001*
Female (*n* = 117)	1–1.159 (0.222)	< 0.001*
Age (*n* = 408)		
Normal (*n* = 50)	0–0.186 (0.142)	
Tumor (21–40, *n* = 27)	0.062–1.066 (0.271)	< 0.003
Tumor (41–60, *n* = 140)	0–1.287 (0.234)	< 0.001*
Tumor (61–80, *n* = 181)	0–1.25 (0.204)	< 0.001*
Tumor (> 80, *n* = 10)	0.028–1.031 (0.424)	0.004*
Race (*n* = 401)		
Normal (*n* = 50)	0–0.363 (0.142)	
Caucasian (*n* = 177)	0–1.159 (0.211)	< 0.001*
African–American (*n* = 17)	0–0.651 (0.237)	0.017*
Asian (*n* = 157)	0–1.394 (0.244)	< 0.001*
Tumor stage (*n* = 390)		
Normal (*n* = 50)	0–0.363 (0.142)	
Stage I (*n* = 168)	0–1.008 (0.176)	< 0.001*
Stage II (*n* = 84)	0.026–1.394 (0.255)	< 0.001*
Stage III (*n* = 82)	0–1.887 (0.374)	< 0.001*
Stage IV (*n* = 6)	0.027–0.581 (0.304)	n.s
Tumor grade (*n* = 407)		
Normal (*n* = 50)	0–0.363 (0.412)	
Grade I (*n* = 54)	0–0.979 (0.222)	0.001*
Grade II (*n* = 173)	0–1.311 (0.214)	< 0.001*
Grade III (*n* = 118)	0–1.268 (0.232)	< 0.001*
Grade IV (*n* = 12)	0.042–1.287 (0.429)	0.017*
Histological subtype (*n* = 421)		
Normal (*n* = 50)	0–0.307 (0.141)	
Hepatocellular carcinoma (*n* = 361)	0–1.268 (0.223)	< 0.001*
Fibrolamellar carcinoma (*n* = 3)	0.055–2.932 (0.979)	0.046*
Hepatocholangiocarcinoma (Mixed) (*n* = 7)	0.13–0.378 (0.142)	n.s

^*^vs. normal

### Association of SAMD13 with expression profiling of tumor microenvironment (TME) in HCC

To further assess the effect of SAMD13 on the TME, we investigated the association between SAMD13 and expression profiling of TME in HCC using the Human Liver Browser and scAtlasLC data sets. It was revealed that SAMD13 was mainly expressed in scar-associated macrophages (SAMs), T cells, tumor-associated macrophages (TAMs), tumor-associated endothelial cells (TECs), and carcinoma cells, while moderately expressed in hepatocytes and cholangiocytes (Fig. 3A, B). Then, we further analyzed between SAMD13 and immune infiltrates in HCC using TIMER database. The results showed that the expression of SAMD13 was significantly correlated with B cell (*r* = 0.159, *p* = 3.19e − 03),CD8 + T cells (*r* = 0.106, *p* = 5.03e-02), CD4 + T cells (*r* = 0.282, *p* = 1.02e − 07), macrophage (*r* = 0.291, *p* = 4.32e-08), neutrophils (*r* = 0.148, *p* = 5.93e − 03), and dendritic cells (*r* = 0.249, *p* = 3.32e-06)in HCC (Fig. 3C). To study the relationship between SAMD13 and infiltrated immune cells, TIMER and GEPIA database were use. Immune gene markers of B cell, T cell (general), CD8 + cell, CD4 + cell, tumor-associated macrophage (TAM), M1 and M2 macrophage, neutrophil, natural killer cell (NK) cell, DC, Th1/2/fh/17cell, Treg, and T cell exhaustion in HCC were assessed. The results revealed that SAMD13 was a significant positive relationship including all immune infiltrates, including B cell (CD17,CD79A), T cell general (CD3D, CD3E, CD2), CD8 + T cell (CD8A, CD8B), CD4 + T cell (CD4), TAM (CD68, IL10), M1 macrophage (IRF5, PTGS2), M2 macrophage (CD163, VSIG4, MS4A4A), neutrophil (ITGAM,CCR7), DC (HLA-DPB1, HLA-DRA, HLA-DPA1, CD1C, NRP1, ITGAX), Th1 (STAT4, STAT1, IFNG, TNF), Th2 (GATA3, STAT6, STAT5A), Th17 (STAT3), Treg (CCR8, TGFB1, STAT5B), and T cell exhaustion (PDCD1, CTLA4, HAVCR2). However, NK cell and Tfh was not significantly associated between SAMD13 and immune infiltrates (Table 2). In addition, the comprehensive prognostic value analysis was performed on SAMD13 expression and immune cell infiltration in HCC. The results of Fig. 3D showed that low expression of SAMD13 along with low immune infiltration of neutrophil, macrophage, macrophage M0, macrophage M2, and myeloid-derived suppressor cells (MDSC) had better prognosis than the high expression of SAMD13 group in HCC. However, there were no significantly changed inCD8 + T cells, CD4 + T cells, B cell, DC, and NK cells (data not shown). These results indicated that SAMD13 is not only closely related to immune cell infiltration, but also correlated with prognosis in HCC.

**Table 2:** Correlation between SAMD13 expression level and gene markers of tumor infiltrating immune cells in TCGA–LIHC

Immune cell	Biomarker	*R* value	*p* value
B cell	CD19	0.15	**
	CD79A	0.1	*
T cell (general)	CD3D	0.19	***
	CD3E	0.17	***
	CD2	0.17	**
CD8^+^ T cell	CD8A	0.13	*
	CD8B	0.14	**
CD4^+^ T cell	CD4	0.14	**
TAM	CCL3	0.058	n.s
	CD68	0.21	***
	IL10	0.22	***
M1 macrophage	NOS2	0.080	n.s
	IRF5	0.28	***
	PTGS2	0.21	***
M2 macrophage	CD163	0.14	**
	VSIG4	0.23	***
	MS4A4A	0.19	***
Neutrophil	CEACAM8	0.04	n.s
	ITGAM	0.28	***
	CCR7	0.12	*
Natural killer cell	KIR2DL4	0.052	n.s
	KIR2DL3	0.005	n.s
	KIR3DL3	−0.02	n.s
	KIR3DL2	0.075	n.s
	KIR2DS4	−0.05	n.s
	KIR2DL1	0.012	n.s
	KIR3DL1	−0.066	n.s
Dendritic cell	HLA–DPB1	0.2	***
	HLA–DQB1	0.073	n.s
	HLA–DRA	0.2	***
	HLA–DPA1	0.2	***
	CD1C	0.2	***
	NRP1	0.22	***
	ITGAX	0.29	***
Th1	TBX21	0.096	n.s
	STAT4	0.19	***
	STAT1	0.23	***
	IFNG	0.11	*
	TNF	0.24	***
Th2	GATA3	0.21	***
	STAT6	0.2	***
	IL13	0.052	n.s
	STAT5A	0.17	***
Tfh	BCL6	0.087	n.s
	IL21	0.021	n.s
Th17	STAT3	0.18	***
	IL17A	0.044	n.s
Treg	FOXP3	0.097	n.s
	CCR8	0.25	***
	TGFB1	0.27	***
	STAT5B	0.15	***
T cell exhaustion	PDCD1	0.14	**
	CTLA4	0.2	***
	LAG3	0.069	n.s
	HAVCR2	0.26	***
	GZMB	−0.015	n.s

^*^P < 0.05

^**^P < 0.01

^***^P < 0.001

**Table 3:** CpGs with methylation across the patients with TCGA–LIHC

CpG	Chromosome	Start	End	UCSC_RefGene_Group	Relation_to_UCSC_CpG_Island	Overall Survival (beta-value) Median Cutoff (50%)		
						HR	95% CI	*p* value
cg20426713	chr1	84297655	84297656	TSS1500	N_Shore	0.86	0.61–1.21	0.386
cg11555919	chr1	84298249	84298250	TSS200	N_Shore	0.8	0.57–1.12	0.196
cg24002839	chr1	84298595	84298596	Body	Island	0.87	0.62–1.23	0.437
cg16026299	chr1	84298692	84298693	Body	Island	1.32	0.94–1.85	0.112
**cg15103960**	chr1	84299021	84299022	Body	Island	**0.71**	0.51–1	**0.052**
**cg02041547**	chr1	84299222	84299223	Body	Island	**0.46**	0.32–0.64	** < 0.001**
**cg23694882**	chr1	84300841	84300842	TSS1500;Body	S_Shore	**1.52**	1.08–2.14	**0.016**
cg17142950	chr1	84301099	84301100	TSS1500;Body	S_Shore	1.04	0.74–1.46	0.838
cg19869443	chr1	84301138	84301139	TSS1500;Body	S_Shore	0.94	0.67–1.33	0.731
**cg23086720**	chr1	84301641	84301642	1stExon;TSS1500;5'UTR;Body	S_Shelf	**0.51**	0.36–0.72	** < 0.001**
cg20466954	chr1	84302676	84302677	1stExon;5'UTR;Body	S_Shelf	0.93	0.66–1.31	0.691
cg13305246	chr1	84303019	84303020	5'UTR;Body	S_Shelf	1.05	0.75–1.48	0.762
**cg15089272**	chr1	84329844	84329845	Body	OpenSea	**1.5**	1.07–2.12	**0.019**
cg22529952	chr1	84347529	84347530	Body	OpenSea	1.27	0.9–1.79	0.171
**cg23925111**	chr1	84350246	84350247	3'UTR	OpenSea	**1.62**	1.15–2.27	**0.006**

CpG (CpG ID), UCSC_RefGene_Name (gene name, based on UCSC annotations); UCSC_RefGene_Group (gene region, based on UCSC annotations); Relation_to_UCSC_CpG_Island (island region, based on UCSC annotations); Regulatory_Feature_Group (type of regulatory region, based on EPIC manifest annotations). Bold font indicates statistical significance

### Association between SAMD13 profiles and clinical characteristics of patients with HCC

**Fig. 4:**
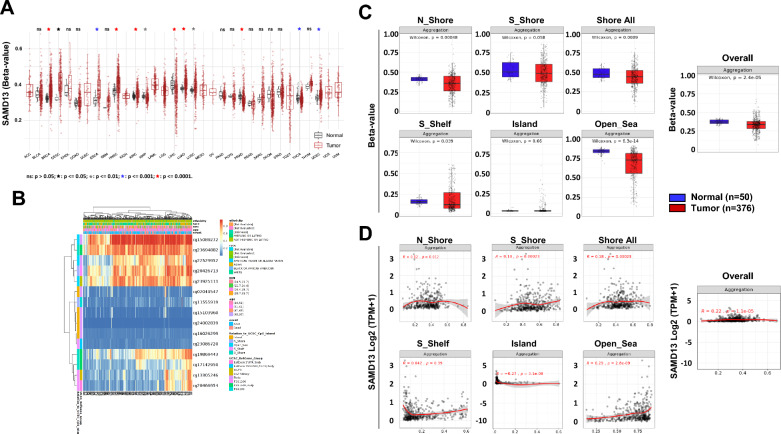
DNA methylation of SAMD13 in HCC of TCGA. **A** The methylation levels of SAMD13 in various types of tumor and their non-tumor tissues in TCGA database. **B** Heatmap of DNA methylation levels of the SAMD13 gene in HCC by MethSurv. **C** Average methylation levels between normal and tumor tissue stratified by genomic location (Wilcoxon rank sum test). **D** Spearman’s correlation between methylation level and mRNA levels of SAMD13 in TCGA–LIHC. N_Shore, North Shore; S_Shore, South Shore; S_Shelf, South Shelf

**Fig. 5:**
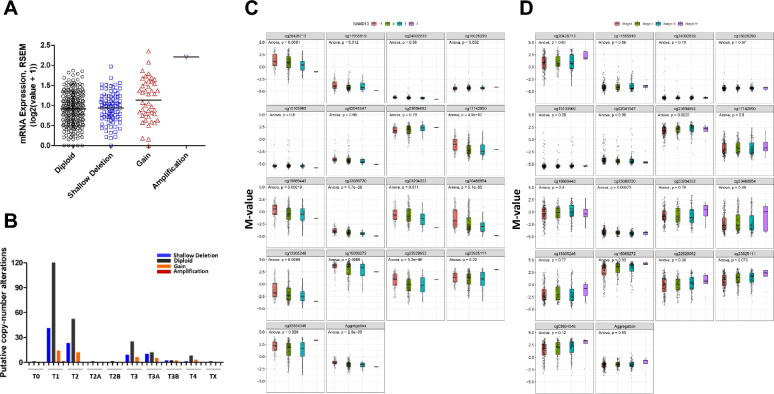
Expression profiles and clinical status of SAMD13 in HCC. **A** Putative copy number alterations and **B** differences in American Joint Committee on Cancer Code staging. Box plot of methylation levels with copy number amplification **C** and tumor stage **D** for SAMD13 in HCC (*M* value, *p* value < 0.05). − 1: single copy deletion; 0: diploid normal copy; + 1: low-level copy number amplification; + 2: high-level copy number amplification

To prove into the role of SAMD13 methylation in HCC, we studied the correlation between SAMD13 methylation state and its expression levels. Based on SMART database, SAMD13 methylation level is significantly lower in HCC tissues compared to normal liver tissues (Fig. 4A) and specific methylation sites were presented in heatmaps using MethSurv database (Fig. 4B). According to the heatmap, there were 15 CpG sites in SAMD13 and we found that average methylation of all CpG sitesof SAMD13 (Overall; Aggregation, *p* = 2.4e-05), including N_Shore (Aggregation, *p* = 0.00048), S_Shore(Aggregation, *p* = 0.058), S_Shelf (Aggregation, *p* = 0.039), and Open_Sea (Aggregation, *p* = 6.3e-14) was significantly lower in tumor tissues than in the normal counterpart (Fig. 4C). At the same time, we performed survival analyses using methylation profiling of SAMD13 CpGs in HCC. The results indicated that CpGs of SAMD13 gene containing cg15103960 (HR, 0.71; 95% CI, 0.51-1; *p* = 0.052), cg02041547 (HR, 0.46; 95% CI, 0.32-0.64; *p* < 0.001), and cg23086720 (HR, 0.51; 95% CI, 0.36-0.72; *p* < 0.001) was associated with better prognosis, while cg23694882 (HR, 1.52; 95% CI, 1.08-2.14; *p* = 0.016), cg15089272 (HR, 1.5; 95% CI, 1.07-2.12; *p* = 0.019), and cg23925111 (HR, 1.62; 95% CI, 1.15-2.27; *p* = 0.006) showed opposite (Table 3). We also found that cg20426713, cg11555919, cg23694882, cg13305246, cg22529952, and cg22529952 probes were significantly lower in tumor tissues than in the normal counterpart, while cg23086720 probe showed higher in tumor compared to normal control (Additional file 1: Figure S2). Given the close relationship between the methylation of SAMD13 and SAMD13 expression, we found a positive correlation between the methylation level and the expression of SAMD13 (Overall; Aggregation, *p* = 1.1e-05), including N_Shore (Aggregation, *p* = 0.012), S_Shore (Aggregation, *p* = 0.00023), and Open_Sea (Aggregation, *p* = 2.8e-09) (Fig. 4D and Additional file 1: Figure S3). Moreover, we examined the copy number alterations and revealed that SAMD13 mRNA had higher copy number gain compare to diploid or shallow deletion (Fig. 5A). Interestingly, later tumor stages including T3, T3A, T3B, and T4 showed a relatively high tendency for deletion or gain of copy number alterations, although absolute copy number alterations were decreased (Fig. 5B). Since the relationship between copy number amplification and individual DNA methylation patterns affects gene regulation, we next explored the relationship between methylation value and copy number amplification in SAMD13. Interestingly, we found that the average methylation value of CpGs of SAMD13 was significantly negative correlation with copy number amplification in HCC (Aggregation, *p* = 2.3e-05) (Fig. 5C). In methylation value on clinical relevance, we found that cg23694882 was much hyper-methylated in stage-dependent manner (*p* = 0.0022), while cg23086720 showed hypo-methylated in stage dependent manner (*p* = 0.00073). These results indicated that methylation value and copy number alterations of SAMD13 gene plays a crucial role in the prognosis that might reflect the complexity/heterogeneity with HCC patients in SAMD13.

### Networks analyses and functional enrichment analyses of SAMD13 in HCC

**Fig. 6:**
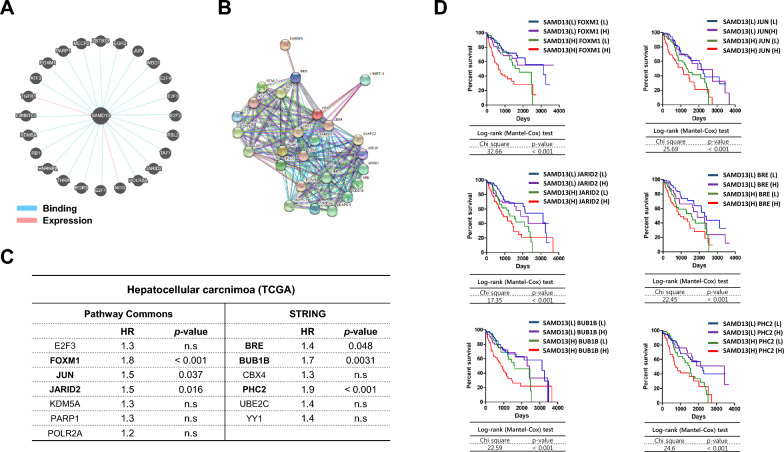
Interaction network and hub proteins. Diagram of potential interactions with SAMD13 by (**A**) Pathways Commons and **B** STRING. **C** Interacting gene lists of statistically significant with SAMD13 expression in HCC tumor group. **D** Kaplan–Meier survival analysis of patients according to SAMD13 and hub genes expression levels. Comparison of patients from TCGA expressing SAMD13 and each hub genes at high or low levels; *p* values derived from the log-rank test are indicated in comparison

 To further investigate the biological role in SAMD13, genes were found to be interacted with SAMD13 based on Pathway Commons and STRING (Fig. 6A, B), and list of genes was presented in Table 4 and 5, respectively. The functions of SAMD13 and interacted genes were classified into each functional group: molecular function, biological process, reactome pathway, and Kyoto Encyclopedia of Genes and Genomes (KEGG) pathway. Among functional and pathway enrichment analysis, SAMD13 was significantly associated with DNA binding on molecular function, including “chromatin binding”, “histone binding”, “methyl-CpG binding”, “general transcription initiation factor binding”; regulation of nucleic acid metabolic process on biological process, including “metaphase/anaphase transition of mitotic cell cycle”, “histone ubiquitination and/or methylation”, “regulation of transcription involved in G1/S transition of mitotic cell cycle”, “hepatocyte apoptotic process”; cell cycle regulation on reactome pathway, including “inactivation of APC/C via direct inhibition of the APC/C complex”, “aberrant regulation of mitotic exit in cancer due to RB1 defects”, “G1 phase”, “oncogene induced senescence”; proteolysis on KEGG pathway, including “ubiquitin mediated proteolysis” and “cell cycle” (Additional file 2: Table S1 and Additional file 3: Table S2). To further explore the potential network co-expressed with SAMD13 gene in HCC, the GEPIA2 database was employed to evaluate the relationship between SAMD13 and co-expressed genes. In the TCGA-HCC dataset, 13 genes were identified with significant correlation with SAMD13 from the tumor group only and a total of six genes including FOXM1, JUN, JARID2, BRE, BUB1B, and PHC2 were shown to significantly predict poor overall survival in log-rank tests in HCC (Fig. 6C). According to the high/low expression groups among these genes combined with SAMD13, we divided the TCGA-HCC samples into four combinations based on the median of gene expression for all six pairs of genes. In survival analysis of the six pairs of gene, the group with high expression of SAMD13 and FOXM1, JUN, JARID2, BRE, BUB1B, or PHC2 had the poorest prognosis, while the groups of low expression of SAMD13 with low expression of all six genes had the best prognosis, respectively (Fig. 6D).

**Table 4:** Correlation analysis between SAMD13 and predicted target gene interactions in Pathways Commons

	Normal				HCC			
	Pearson correlation	*p* value	Spearman correlation	*p* value	Pearson correlation	*p* value	Spearman correlation	*p* value
ATF2	0.24	n.s	0.34	0.015	0.25	< 0.001	0.25	< 0.001
E2F1	0.1	n.s	0.14	n.s	0.053	n.s	0.18	< 0.001
E2F2	0.23	n.s	0.33	0.019	0.24	< 0.001	0.31	< 0.001
**E2F3**	0.03	n.s	0.15	n.s	0.24	< 0.001	0.35	< 0.001
E2F4	0.47	< 0.001	0.37	0.089	0.42	< 0.001	0.31	< 0.001
EGR2	−0.0039	n.s	0	n.s	0.094	n.s	0.2	< 0.001
EGR3	0.17	n.s	0.25	n.s	0.08	n.s	0.2	< 0.001
**FOXM1**	0.072	n.s	0.22	n.s	0.25	< 0.001	0.31	< 0.001
HNRNPL	0.37	0.0078	0.36	0.01	0.42	< 0.001	0.34	< 0.001
**JARID2**	0.095	n.s	0.17	n.s	0.28	< 0.001	0.31	< 0.001
**JUN**	0.074	n.s	0.062	n.s	0.2	< 0.001	0.23	< 0.001
NOG	0.11	n.s	0.29	0.044	0.14	0.009	0.13	0.0095
**KDM5A**	0.19	n.s	0.26	n.s	0.33	< 0.001	0.22	< 0.001
L3MBTL3	0.32	0.025	0.37	0.0081	0.21	< 0.001	0.24	< 0.001
MBD1	0.36	0.0097	0.39	0.0051	0.37	< 0.001	0.31	< 0.001
MECP2	0.45	0.0011	0.36	0.01	0.19	< 0.001	0.22	< 0.001
**PARP1**	0.057	n.s	0.14	n.s	0.19	< 0.001	0.28	< 0.001
**POLR2A**	0.12	n.s	0.18	n.s	0.22	< 0.001	0.23	< 0.001
RB1	0.26	n.s	0.36	0.01	0.28	< 0.001	0.25	< 0.001
RBL2	0.19	n.s	0.31	0.031	0.15	0.0041	0.091	n.s
TAF1	0.28	n.s	0.33	0.018	0.25	< 0.001	0.21	< 0.001
THRA	0.43	0.002	0.3	0.032	0.25	< 0.001	0.24	< 0.001
ZBTB33	0.14	n.s	0.28	0.049	0.059	n.s	0.16	0.0021
TGFB1	0.46	< 0.001	0.34	0.017	0.17	0.0013	0.27	< 0.001

Bold font indicates statistical significance in HCC group only

**Table 5:** Correlation analysis between SAMD13 and predicted target gene interactions in STRING

	Normal				HCC			
	Pearson correlation	*p* value	Spearman correlation	*p* value	Pearson correlation	*p* value	Spearman correlation	*p* value
ANAPC1	0.32	0.022	0.39	0.0051	0.31	< 0.001	0.29	< 0.001
ANAPC11	0.23	n.s	0.18	n.s	0.099	n.s	0.063	n.s
ANAPC2	0.28	0.047	0.2	n.s	0.21	< 0.001	0.15	0.0038
ANAPC5	0.45	< 0.001	0.36	0.011	0.29	< 0.001	0.31	< 0.001
ASXL3	−0.032	n.s	0.023	n.s	-0.032	n.s	0.016	n.s
BARD1	0.022	n.s	0.28	0.047	0.27	< 0.001	0.34	< 0.001
**BRE**	0.22	n.s	0.18	n.s	0.29	< 0.001	0.32	< 0.001
**BUB1B**	0.1	n.s	0.21	n.s	0.27	< 0.001	0.34	< 0.001
CBX2	0.37	0.0081	0.44	0.0016	0.34	< 0.001	0.27	< 0.001
**CBX4**	0.27	n.s	0.2	n.s	0.27	< 0.001	0.2	< 0.001
CBX8	0.29	0.043	0.26	n.s	0.41	< 0.001	0.26	< 0.001
CDC16	0.33	0.018	0.38	0.0069	0.24	< 0.001	0.28	< 0.001
EED	0.47	< 0.001	0.47	< 0.001	0.32	< 0.001	0.33	< 0.001
EZH2	0.33	0.02	0.39	0.0051	0.24	< 0.001	0.35	< 0.001
HNRNPL	0.37	0.0078	0.36	0.01	0.42	< 0.001	0.34	< 0.001
L3MBTL3	0.32	0.025	0.37	0.0081	0.21	< 0.001	0.24	< 0.001
PCGF1	0.44	0.0013	0.3	0.032	0.28	< 0.001	0.24	< 0.001
**PHC2**	−0.0026	n.s	−0.0072	n.s	0.4	< 0.001	0.35	< 0.001
SCML2	0.2	n.s	0.21	n.s	0.086	n.s	0.19	< 0.001
SUZ12	0.27	n.s	0.38	0.0068	0.28	< 0.001	0.3	< 0.001
**UBE2C**	0.14	n.s	0.24	n.s	0.2	< 0.001	0.24	< 0.001
UBE2S	0.37	0.0085	0.33	0.02	0.23	< 0.001	0.28	< 0.001
**YY1**	0.17	n.s	0.26	n.s	0.26	< 0.001	0.27	< 0.001
ZFP42	NA	NA	NA	NA	0.15	0.0032	0.16	0.0015

Bold font indicates statistical significance in HCC group only

### Association of SAMD13 expression with the acquired drug resistance and immune checkpoint blockade (ICB) genes

**Fig. 7:**
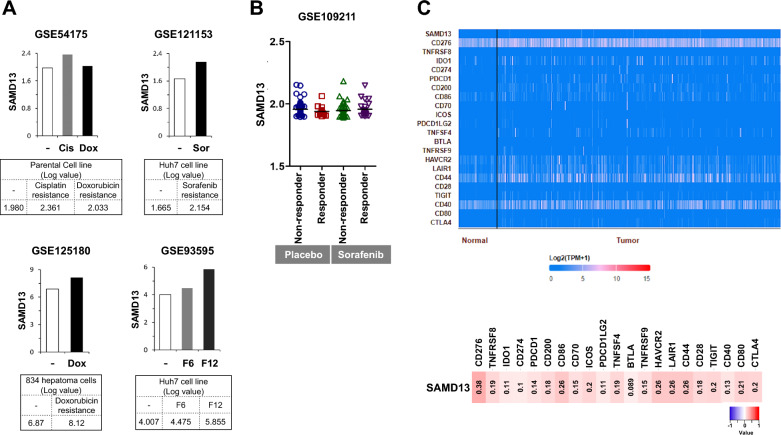
SAMD13 is upregulated in chemotherapeutic drug-resistant HCC. **A** Expression of SAMD13 in cisplatin- or doxorubicin-resistant (GSE54175), sorafenib-resistant (GSE121153), doxorubicin-resistant (GSE125180), and JNJ-28841072-resistant cells (GSE93595) was shown by analyzing the GEO database. **B** SAMD13 levels was determined in HCC patients responding or non-responding to sorafenib treatment or not.–, parent cells; Cis, in vitro Cisplatin-resistant subclones; Dox, in vitro Doxorubicin-resistant subclones; Sor, in vitro Sorafenib-resistant subclones; F6, in vivo drug-tolerant subclones (6 months); F12, in vivo drug-tolerant subclones (12 months). **C** Heat-map plot of Spearman’s correlation between SAMD13 and ICB related genes from TCGA–LIHC data set

Since the biological role of SAMD13 enriches the “cell cycle” and “nucleic acid metabolic process”, we further examined the chemosensitivity to cisplatin, doxorubicin, sorafenib, and JNJ-28841072 which have been shown to potently inhibit the cell cycle used for the treatment of HCC from three independent GEO data set, including GSE54175 (identification of chemo-resistant genes in human metastatic HCC), GSE121153 (sorafenib-resistant HCC), GSE125180 (doxorubicin-resistant HCC), and GSE93595 (anti-angiogenic drug resistant HCC). As shown Fig. 7A, SAMD13 mRNA level was elevated in almost all cell lines with conventional chemotherapy, based on analyses from the GEO databases. Typically, SAMD13 was positively correlated with the elevation of gene expression for cisplatin-resistant MHCC97L cells (GSE54175), sorafenib-resistant Huh7 cells (GSE121153), doxorubicin-resistant 834 cells (GSE125180), and JNJ-28841072-resistant Huh7 cells which had acquired resistance under long-term anti-angiogenic therapy from serial transplantation in immunocompromised mice (GSE93595). To further verify the clinical significance of SAMD13 expression for assessing response of chemotherapy, drug-cancer response data set was investigated. One cohort (GSE109211) treated with placebo or sorafenib was retrieved, which contained 140 patients randomized to receive 98 non-responders and 42 responders. Compared to placebo- or sorafenib-treatment, there was no significant differences between responders and non-responders (Fig. 7B), as well as recurrence of HCC (Additional file 1: Figure S4). Since the role of SAMD13 in ICB treatment of HCC has not been reported, we further assessed the TCGA–LIHC data to explore the correlation between SAMD13 and some ICB related genes. The results demonstrated that SAMD13 was significantly positive correlation with ICB genes, including CD276, CD86, ICOS, HAVCR2, LAIR1, CD44, TIGIT, CD80, CTLA4 (Fig. 7C). Collectively, these results suggest that SAMD13 expression might not be involved in chemotherapy response to cancer treatment, but rather a target for ICB treatment that can counter acquired chemical resistance.

## Discussion

HCC accounts for more than 90% of primary liver tumors and the 5-year survival rate of HCC is only 18% [26, 27]. The main reason for rapid progression and high mortality of HCC leads to treatment failure caused by resistance to conventional chemotherapy, recurrence, and metastasis in patients with HCC, and eventually shows a worse prognosis. Hence, the discovery of promising biomarkers for clinical diagnosis and treatment of patients with HCC is urgently needed.

In this study, we evaluated the expression, methylation, drug-resistance, and prognostic significance of SAMD13 as a new prognostic biomarker in HCC based on bioinformatics approaches. Our result revealed that SAMD13 was significantly overexpressed in TCGA including HCC and we validated using three independent GEO data sets (GSE22058, GSE25097, and GSE45436). Although SAMD13 is highly expressed in various types of cancer, it was significantly associated with shorter OS, DSS, DFI, and PFI in patients with HCC, even poorly differentiated HCC cell lines which tend to be more aggressive and higher grade. SAMD13 could be attributed to various immune infiltration events, such as B cells, T cells, macrophages, neutrophil, and DCs. In analysis of methylation profiling, CpGs of SAMD13 gene containing cg15103960, cg02041547, cg23694882, cg23086720, cg15089272, and cg23925111 was associated with prognosis, and SAMD13 gene showed hypomethylation. In networks and functional enrichment analyses, six hub genes (FOXM1, JUN, JARID2, BRE, BUB1B, and PHC2) which were up-regulated in HCC tissues in comparison with normal tissues. Meanwhile, high-level of six-hub gene expressions and high-level SAMD13 expression were significantly associated with the worst OS in correlation analysis. More importantly, SAMD13 expression could be a potential for immuno-checkpoint inhibitor (ICI) treatment, although it exhibits acquired diverse anti-cancer drug resistant in HCC. Taken together, these results suggested that SAMD13 play an important role in poor clinical outcome and additional genetic and/or epigenetic alterations of SAMD13 gene promotes the development of HCC. To our knowledge, this is report to demonstrate that SAMD13 is the novel biomarker to further clarify the prognostic factors for HCC.

SAMD13 is known a protein coding gene which enables chromatin and histone binding activity involved in negative regulation of transcription. Only one study showed that SAMD13 is negatively associated with invasive micropapillary carcinoma of breast cancer, especially micropapillary area [13], but its clinical impact on cancers remains totally unclear, even contrary. In this study, our findings revealed that the level of SAMD13 expression was significantly up-regulated in the tumor group compared to the non-tumor group, while high level of SAMD13 expression was significantly associated with the worst prognosis in HCC. As high-grade (poorly differentiated) cancer cells tended to be more motile and aggressive than well-differentiated cancer cells [28], we confirmed that mRNA expression of SAMD13 in well- and poorly differentiated HCC cell lines. We also indicated that SAMD13 was positively associated with immune cell infiltration. Since immune cells and stromal cells represent typical TME, their interactions are related to the tumor invasion, recurrence, metastasis, and the effect of immunotherapy, and prognosis [29–31]. Besides, Pappas et al. revealed that SAMD9L plays a role in the migration of INF-beta affected T cells [32]. In the present study, the expression levels of SAMD13 exhibited a positive correlation with diverse immune cells, including B cell, T cell, TAM, macrophage, neutrophil, and DCs. Interestingly, low SAMD13 expression was associated with low immune cell infiltration of neutrophil, macrophage, macrophage M0/M2, and MDSC, it showed better prognosis (Fig. 3D). In patients with HCC, high levels of neutrophil, macrophage (tumor-derived macrophage or macrophage M2), and MDSC are reported to be associated with poor survival [33–35], it is hypothesized that SAMD13 may play an important role on the TME in HCC and further study of the immune microenvironment and HCC feature should be addressed. In epigenetic analysis, methylation in SAMD13 affected prognosis and copy number alterations, especially for some specific CpG sites. Methylation of cytosine residues in CpG dinucleotide pairs could serve as not only prognostic biomarkers but also therapeutic targets [36–40]. Because aberrant DNA methylation contribute directly to dysregulation of gene expression, such as oncogenes or tumor suppressor genes, respectively [41–43]. Since newly discovered six CpGs located in genes were able to predict HCC OS, CpG methylation of SAMD13 gene could provide a new prognostic clue in HCC.

In addition, using Pathway Commons and STING, we found that thirteen genes had significantly interacted with SAMD13 and finally identified six genes positively correlated with SAMD13 expression of HCC–FOXM1, JUN, JARID2, BRE, BUB1B, and PHC2. Among these six genes, FOXM1, which is a transcription factor fork-head box M1, has been proved to play a role in tumor progression and poor prognosis in HCC [44, 45]. JUN has been reported to be overexpressed in diverse cancers including HCC and can play an oncogenic transcription factor [46, 47]. JARID2 is a DNA-binding protein which plays an essential role in transcriptional regulation during embryonic development [48, 49]. Recently some researchers reported that JARID2 can promote invasion and metastasis and was correlated with worse OS with HCC [50, 51]. In HCC, the over expressed BRE which is core component of the deubiquitin complex. BRCA1-A and BUB1B belong to core elements of the spindle assembly checkpoint (SAC) correlated with tumor progression, worse OS and DFS in HCC [52–55]. The role of PHC2, which is a transcriptional repressor of target genes by mainly modulating histone methylation, has not yet been fully understood in HCC. Of note, SAMD13 and its associated six-hub gene identified as having transcriptional and epigenetic regulation in networks analyses and functional enrichment analyses resulted in poor progression in patients with HCC. In summary, our study provides clues regarding the biological roles and molecular characterization of these genes with SAMD13, and it could act as new prognostic biomarkers for HCC.

Furthermore, we demonstrated that upregulation of SAMD13 expression could act as acquired drug resistance, as well as a potential for immune checkpoint targeted therapy. Although conventional chemotherapies including targeted therapies based on tyrosine kinase inhibitors (TKIs) and conventional chemotherapy are in clinical use, the reasons for the high resistance in HCC are not fully understood. However, the proposed mechanisms of drug resistance are associated with cell cycle regulation, DNA damage repair, drug detoxification, and apoptotic pathway, and epigenetic functions such as DNA methylation which includes certain genes that play a role [56, 57]. Since we observed high SAMD13 expression in cisplatin-, sorafenib-, doxorubin- and JNK-28841072 resistant HCC sublines in the GEO database, this suggests that SAMD13 is likely to mediate acquired drug resistance. All these indicate that the biological role of SAMD13 enriched in “regulation of nucleic acid metabolic process” and “cell cycle regulation” participates in acquired chemotherapy resistance and translational process, further supporting a role for CpG methylation of SAMD13 linked with worse prognosis in patients with HCC. We also evaluated the relationship between SAMD13 and ICB related genes in HCC. As a result, SAMD13 showed significantly positive correlations with multiple immunotherapy-predicted pathways and checkpoints, which could affect the efficacy of ICB. Therefore, the SAMD13-based prediction model might provide individualized precision treatment to patients and further study should be addressed to confirm these results.

## Conclusion

Our bioinformatics analysis identified that SAMD13 may be critical in the development and prognosis of HCC. However, our study was performed based on bioinformatic analysis, further experimental validations should be addressed to confirm the results of this prediction in HCC. We hope present study can provide some evidence for the potential use as cancer therapeutic and prognostic biomarker of HCC from new insights.

### Supplementary Information

**Additional file 1: Figure S1.** Pan-cancer view of SAMD13 gene expression in GENT2 database. **Figure S2.** Scatterplot of DNA methylation levels of SAMD13 in samples of normal and HCC. The probes located in CpG N_Shore (A), S_Shore (B), S_Shelf (C), Open_Sea (D), and Island (E), respectively. **Figure S3.** Spearman’s correlation analysis between each methylation level and mRNA levels of SAMD13 gene in TCGA–LICH. The probes located in CpG N_Shore (A), S_Shore (B), S_Shelf (C), Open_Sea (D), and Island (E), respectively. **Figure S4.** Relevance of SAMD13 gene to patient HCC recurrence in GSE76427 (A) and TCGA–LIHC (B). The OSdream database (https://bioinfo.henu.edu.cn/OSdream/OSdream.html) was used to predict the SAMD13 gene and HCC recurrence.

**Additional file 2. **Functional and pathway enrichment analysis of SAMD13 on Pathway Commons including molecular function, biological process, and reactome pathway.

**Additional file 3. **Functional and pathway enrichment analysis of SAMD13 on STRING including molecular function, biological process, reactome pathway, and KEGG pathways.

## Data Availability

Not applicable.
