# Supplementary material for: SAMD13 serves as a useful prognostic biomarker for hepatocellular carcinoma
Source: Eur J Med Res. 2023 Nov 15;28:514. doi: 10.1186/s40001-023-01347-5 (PMC10648382; doi:10.1186/s40001-023-01347-5)

Additional file Fig. 1 Pan-cancer view of SAMD13 gene expression in GENT2 database

Additional file Fig. 2 Scatterplot of DNA methylation levels of SAMD13 in samples of normal and HCC. The probes located in CpG N_Shore (A), S_Shore (B), S_Shelf (C), Open_Sea (D), and Island (E), respectively.


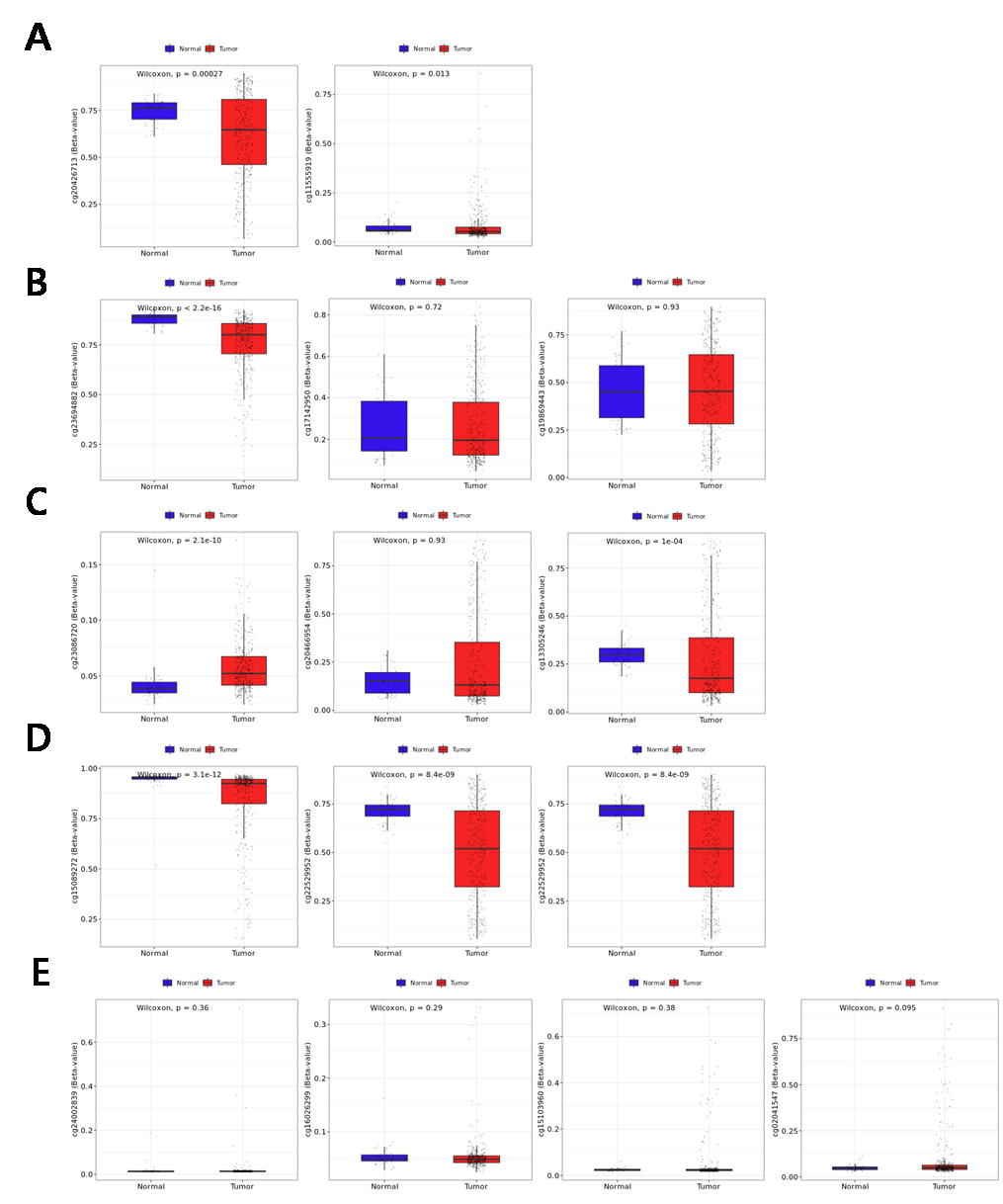


Additional file Fig. 3 Spearman’s correlation analysis between each methylation level and mRNA levels of SAMD13 gene in TCGA-LICH. The probes located in CpG N_Shore (A), S_Shore (B), S_Shelf (C), Open_Sea (D), and Island (E), respectively.


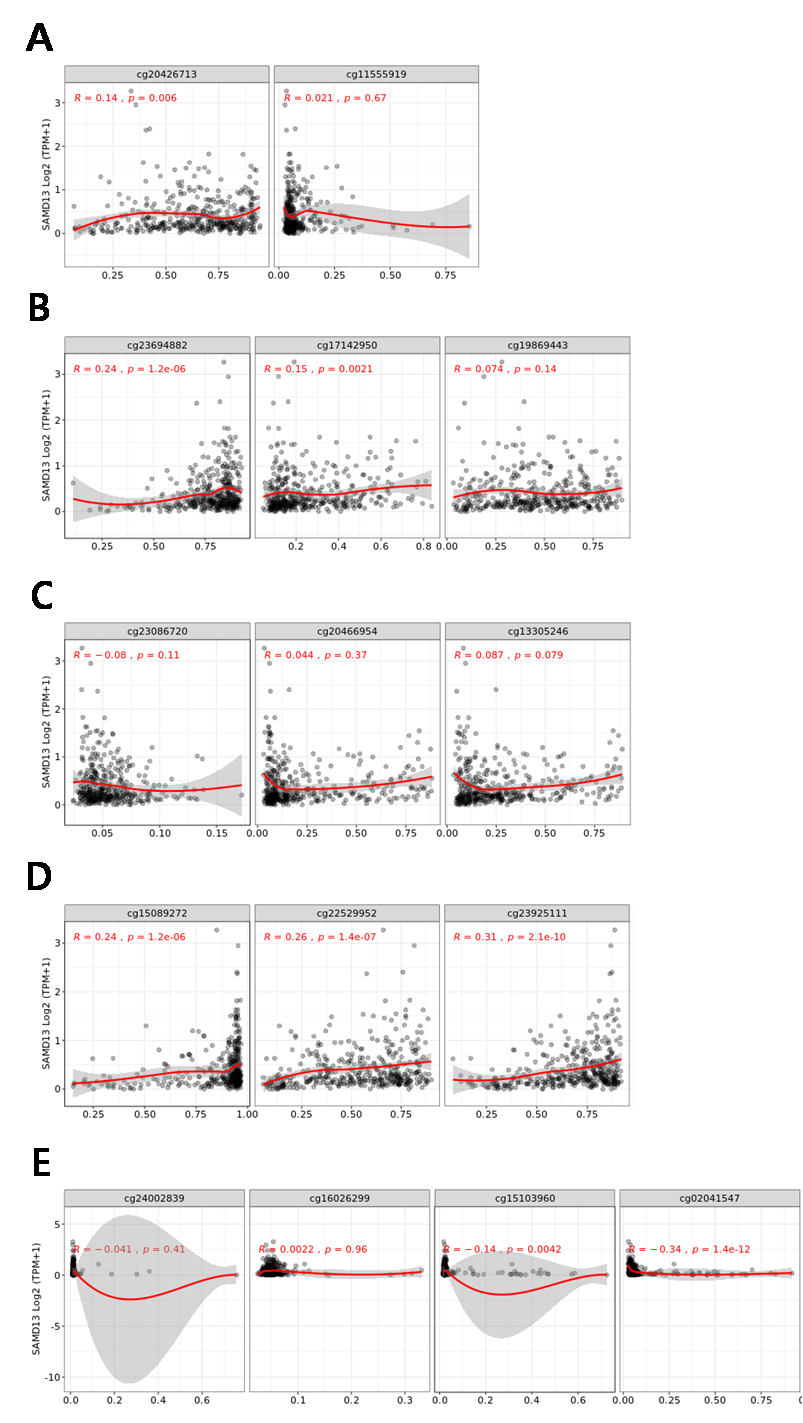


Additional file Fig. 4 Relevance of SAMD13 gene to patient HCC recurrence in GSE76427 (A) and TCGA-LIHC (B). The OSdream database (https://bioinfo.henu.edu.cn/OSdream/OSdream.html) was used to predict the SAMD13 gene and HCC recurrence.


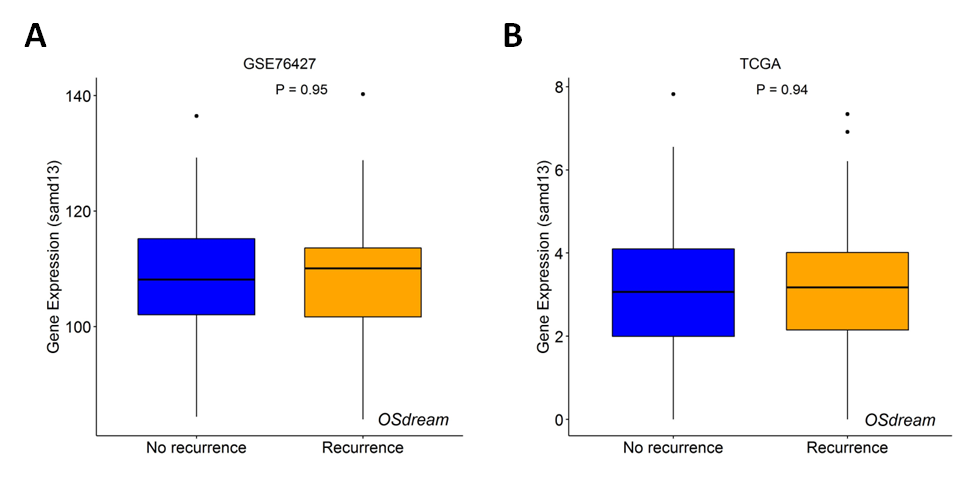

Supplement: Supplementary file 1 — Additional file 1: Figure S1. Pan-cancer view of SAMD13 gene expression in GENT2 database. Figure S2. Scatterplot of DNA methylation levels of SAMD13 in samples of normal and HCC. The probes located in CpG N_Shore (A), S_Shore (B), S_Shelf (C), Open_Sea (D), and Island (E), respectively. Figure S3. Spearman’s correlation analysis between each methylation level and mRNA levels of SAMD13 gene in TCGA–LICH. The probes located in CpG N_Shore (A), S_Shore (B), S_Shelf (C), Open_Sea (D), and Island (E), respectively. Figure S4. Relevance of SAMD13 gene to patient HCC recurrence in GSE76427 (A) and TCGA–LIHC (B). The OSdream database (https://bioinfo.henu.edu.cn/OSdream/OSdream.html) was used to predict the SAMD13 gene and HCC recurrence. [file 40001_2023_1347_MOESM1_ESM.docx]
